# Supplementary material for: Gut Microbiome and Its Cofactors Are Linked to Lipoprotein Distribution Profiles
Source: Microorganisms. 2022 Oct 31;10(11):2156. doi: 10.3390/microorganisms10112156 (PMC9699503; doi:10.3390/microorganisms10112156)
Supplement: Supplementary file 1 [file microorganisms-10-02156-s001.zip › microorganisms-1966304-supplementary.pdf]

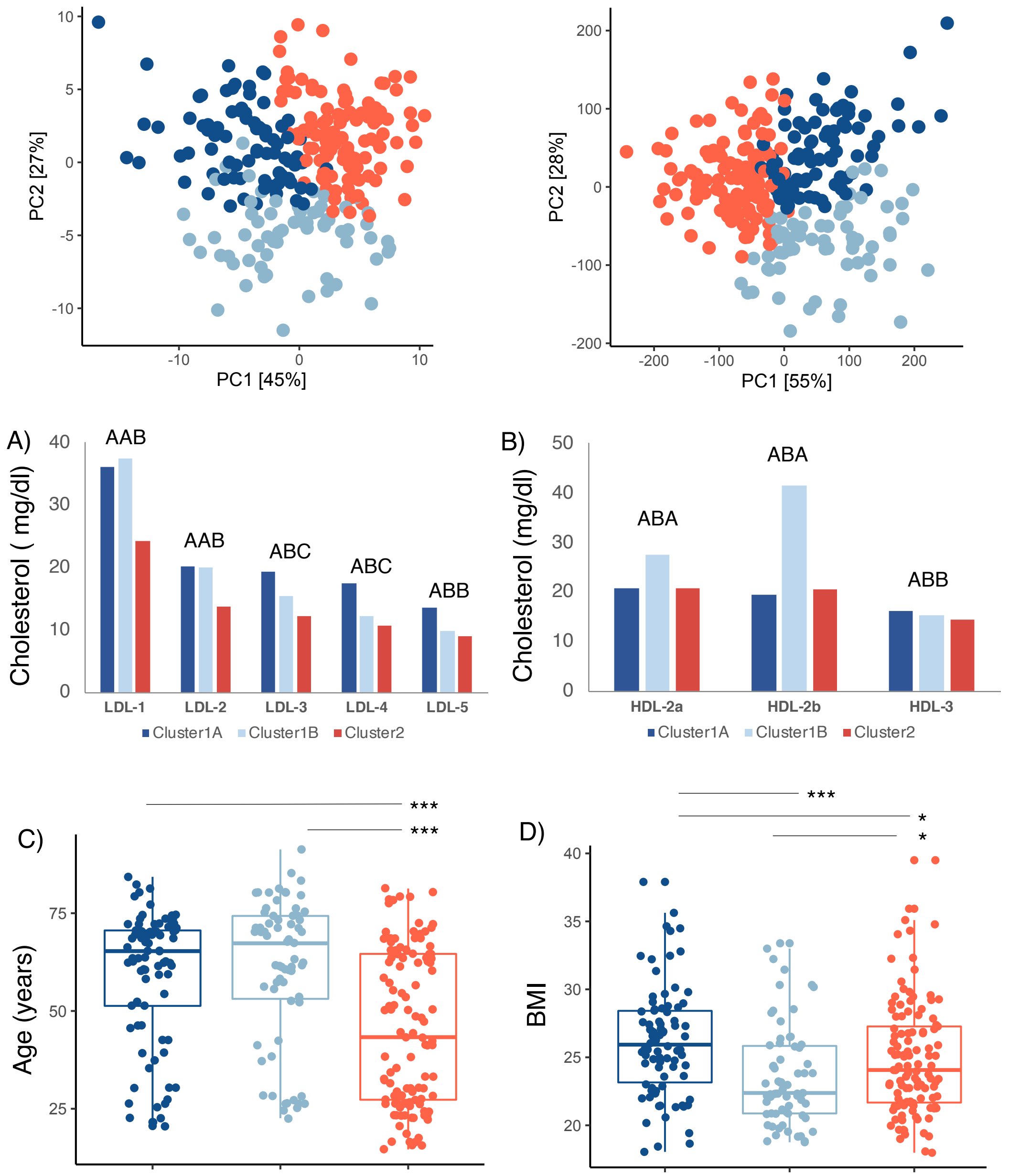

Supplementary Figure S1. Cholesterol sub-fractions distribution and covariates adjusted by sex effect.
Principal Component Analysis (PCA) discriminates clusters based on **A**) scaled and **B**) non-scale lipoproteins data. Average concentrations of circulating **C**) LDL and **D**) HDL sub-fractions among LPD clusters. Multiple comparisons ($P \leq 0.05$) were carried out by Tukey's HSD and display by letters A, B and C. Distribution of **E**) age and **F**) body mass index (BMI) between subjects belonging to C1A, C1B and C2 a. Stars show statistical level of significance (*$p \leq 0.05$, ***$p \leq 0.001$)

A)

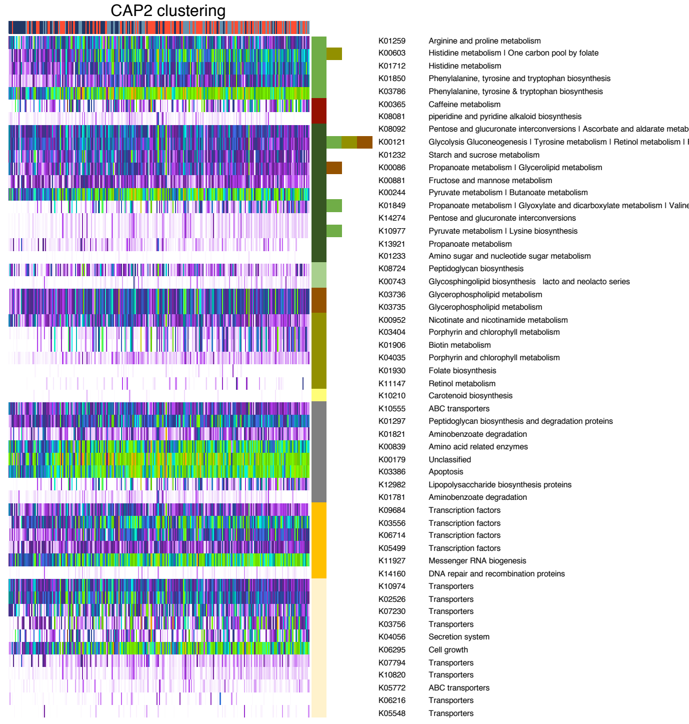

Supplementary Figure S2. zOTUs and KO functions at 3rd level KEGG

A) Overview of selected KOs-PICRUSt (extension of Fig. 2B) displaying functional traits at 3rd level KEGG.

B) Spearman's rank correlations between zOTUs and LPDs sub-fractions (extension of Fig. 2G).

C) Spearman's rank correlations between KOs-PICRUSt and LPDs sub-fractions (extension of Fig. 2H) displaying functional traits at 3rd level KEGG.

B)

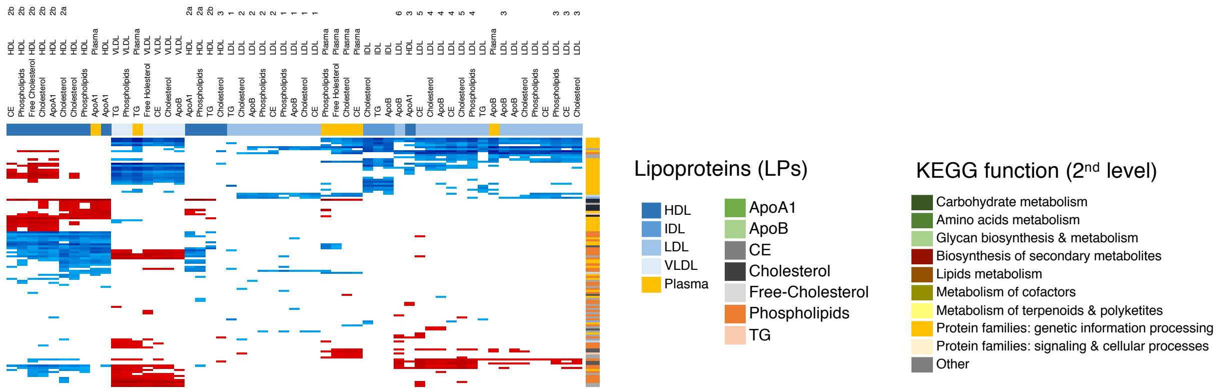

C)

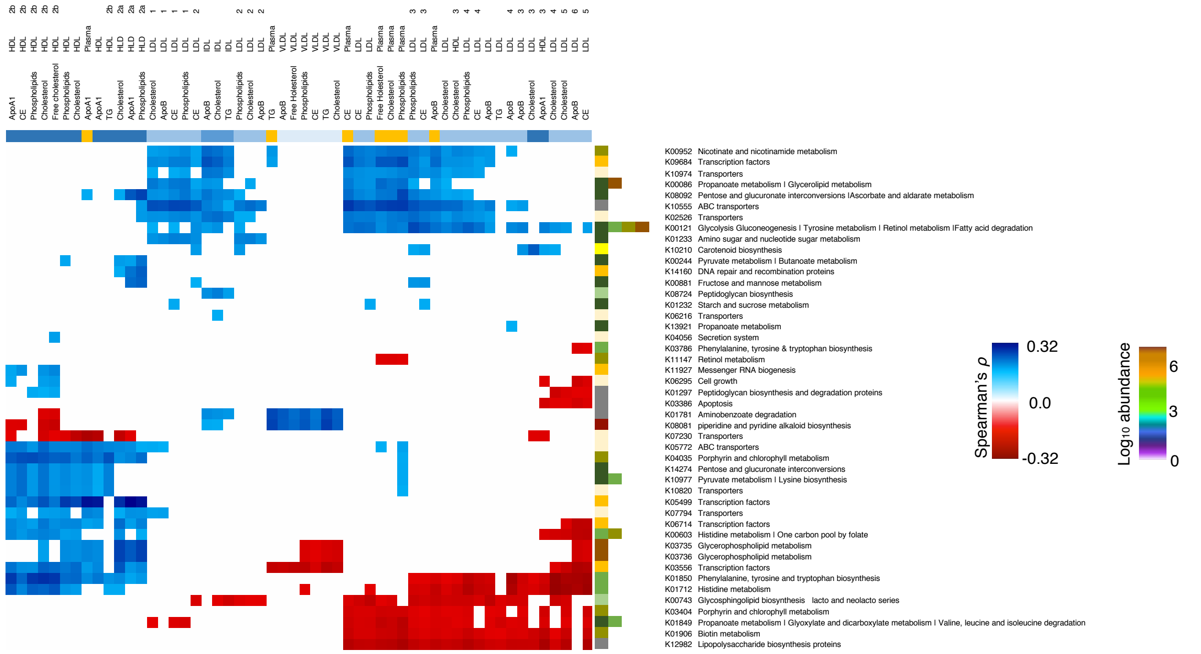

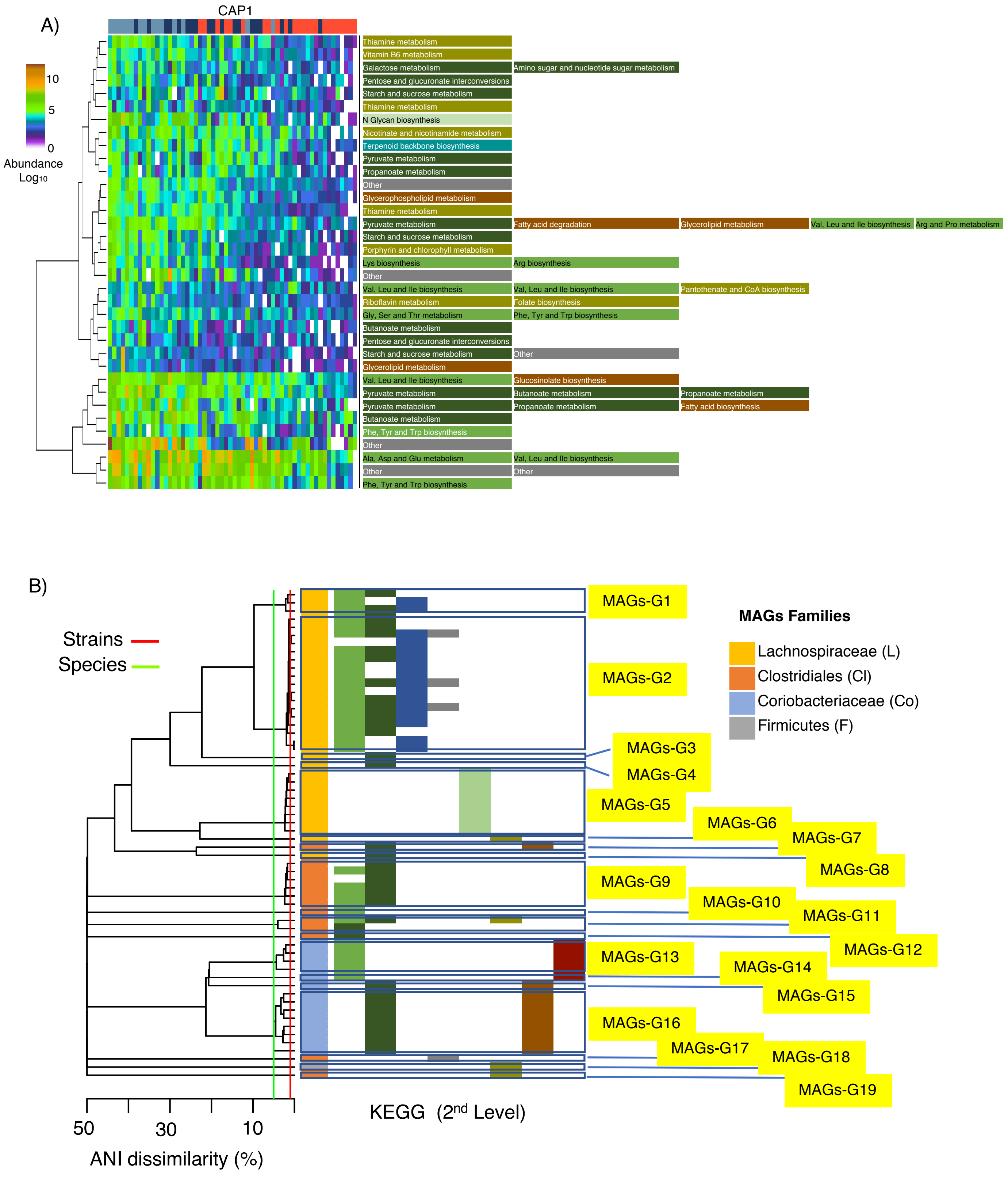

Supplementary Figure S3. KO functions (3rd level KEGG) linked to MAGs

A) Overview of most discriminatory (extension of Fig. 5B) KOs summarized to their 3rd level KEGG function.

B) Phylogeny of MAGs outlined into species groups (95 ANI) – (extension of Fig. 5D)

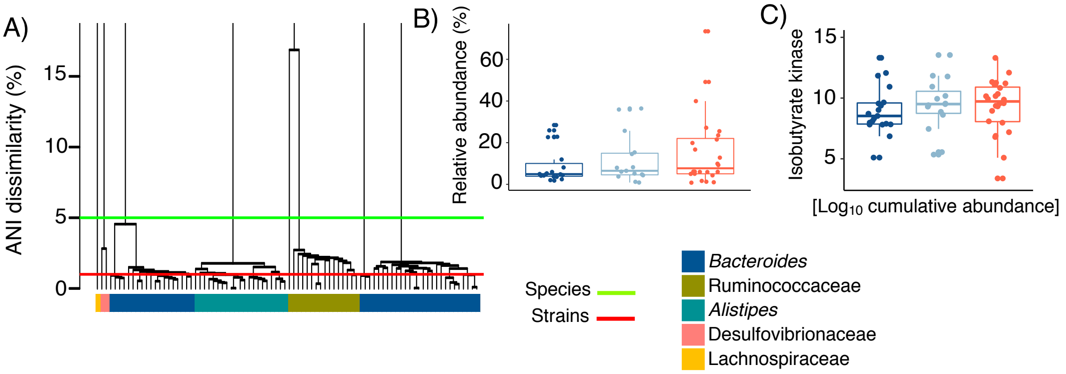

Supplementary Figure S4. Isobutyrate kinase contributing MAGs

A) Phylogeny of MAGs outlined into species groups (95 ANI) containing isobutyrate kinase genes.

B) Cumulative relative distribution MAGs within LPDs clusters.

C) Abundance of branched-chain fatty acid (BCFA) genes kinase within LPDs clusters.
